# Anodal Transcranial Direct Current Stimulation Provokes Neuroplasticity in Repetitive Mild Traumatic Brain Injury in Rats

**DOI:** 10.1155/2017/1372946

**Published:** 2017-07-09

**Authors:** Ho Jeong Kim, Soo Jeong Han

**Affiliations:** ^1^Department of Rehabilitation Medicine, Seonam Hospital, Ewha Womans University Medical Center, Seoul, Republic of Korea; ^2^Department of Rehabilitation Medicine, School of Medicine, Ewha Womans University, Seoul, Republic of Korea

## Abstract

Repetitive mild traumatic brain injury (rmTBI) provokes behavioral and cognitive changes. But the study about electrophysiologic findings and managements of rmTBI is limited. In this study, we investigate the effects of anodal transcranial direct current stimulation (tDCS) on rmTBI. Thirty-one Sprague Dawley rats were divided into the following groups: sham, rmTBI, and rmTBI treated by tDCS. Animals received closed head mTBI three consecutive times a day. Anodal tDCS was applied to the left motor cortex. We evaluated the motor-evoked potential (MEP) and the somatosensory-evoked potential (SEP). T2-weighted magnetic resonance imaging was performed 12 days after rmTBI. After rmTBI, the latency of MEP was prolonged and the amplitude in the right hind limb was reduced in the rmTBI group. The latency of SEP was delayed and the amplitude was decreased after rmTBI in the rmTBI group. In the tDCS group, the amplitude in both hind limbs was increased after tDCS in comparison with the values before rmTBI. Anodal tDCS after rmTBI seems to be a useful tool for promoting transient motor recovery through increasing the synchronicity of cortical firing, and it induces early recovery of consciousness. It can contribute to management of concussion in humans if further study is performed.

## 1. Introduction

Mild traumatic brain injury (mTBI) or concussion is an acute closed head injury resulting from external physical force applied to the head. According to the operational definition provided by the WHO collaborating task force team, mTBI could lead to confusion or disorientation and it could be symptomized by loss of consciousness for 30 minutes or less or posttraumatic amnesia for less than 24 hours. The Glasgow Coma Scale score is described as 13–15 after 30 minutes postinjury or later upon presentation for health care [1, 2]. In repetitive brain injury, long-term neurological impairment presents as memory disturbance, parkinsonism, behavioral abnormalities, personal changes, speech irregularities, and gait abnormalities [3]. Pathologic changes after repetitive mTBI (rmTBI) have been reported, including brain volume loss, tau-immunoreactive neurofibrillary tangles, the hallmark of chronic traumatic encephalopathy, and amyloid *β* deposition, the hallmark of Alzheimer's disease [4–6]. In a previous rodent study about rmTBI, not only behavioral changes but also pathologic changes were well proven. Behavioral changes included prolonged duration of righting reflex, decreased balance and motor coordination, and decreased spatial learning and memory. Pathologic changes include chronic gliosis, multifocal axonopathy, neurodegeneration, ventriculomegaly, and cortical thinning [7–10]. However, there is no study assessing the electrophysiological changes after rmTBI, and studies on its management are rather scarce.

Transcranial direct current stimulation (tDCS) is used to polarize local brain regions by the noninvasive application of weak direct current. The mechanism of action is thought to be associated with changes in the resting membrane potential of a neuron led by constant gradient voltage that induces ionic currents. Sodium and calcium channels are modulated by delivering subthreshold electrical currents to the brain. The direction of the current that is applied by tDCS affects the outcome. Cathodal tDCS decreases cortical excitability, whereas anodal tDCS increases cortical excitability [11, 12]. Previous studies have reported that anodal tDCS provides a therapeutic effect in patients with neurologic disorders, such as stroke, Parkinson's disease, and Alzheimer's disease [13–15]. A recent study showed that anodal tDCS improves spatial memory during the early stage of traumatic brain injury in rats [16]. But, there is still no study assessing the effect of anodal tDCS on rmTBI.

Righting reflex was defined as the animal's ability to right itself from a supine to a prone position. In animals, delayed recovery of the righting reflex indirectly means prolonged loss of consciousness. And previous study showed prolonged righting reflex after repetitive mild traumatic brain injury [8, 10].

The purpose of this study is to investigate the electrophysiological, histologic, and behavioral changes after rmTBI and to reveal the effect of anodal tDCS treatment on rmTBI in a rat model.

## 2. Materials and Methods

### 2.1. Experimental Design

A total of thirty-one male Sprague Dawley rats (postnatal day forty-two, 180–240 g) were housed in laboratory cages under a controlled environment (21.0–24.0°C) and maintained in a 12/12 hour light/dark cycle with food and water ad libitum. All experimental protocols were approved by our Institutional Animal Care and Use Committee. Previous study suggested that postnatal day 30 in rat was roughly equivalent to late childhood, 7–11 years of age [17]. Metabolic developmental profiles showed that postnatal day 35 rats reach roughly 90% of adult values and sexual maturity is completed at postnatal day 60 [7]. Based on previous studies, postnatal day 42 meant late juvenile to early adult which is the common age of mild traumatic brain injury (teenagers and young adult) [3]. All procedures and evaluations were carried out under anesthesia. Anesthesia with Zoletil® (tiletamine/zolazepam, 15 mg/kg) was administered via an intramuscular injection. Animals were assigned to the sham group (*n* = 10), the rmTBI group (*n* = 11), and the anodal tDCS group (*n* = 10). The animals in the sham group were given only anesthesia without head impact. In the rmTBI and anodal tDCS groups, closed head traumatic brain injury was repeated three consecutive times in a single day. Then, anodal tDCS was applied in the anodal tDCS group only. The rats in all groups were then placed in a supine position and monitored for the righting reflex time. Motor-evoked potential and sensory-evoked potential tests were performed at baseline and after all procedures in each group such as rmTBI, anodal tDCS treatment, or noninjury. Brain MRI was performed 12 days after rmTBI. Five rats in each group were sacrificed at 12 days after rmTBI or sham injury for immunohistochemical analysis.

### 2.2. The rmTBI Model

rmTBI was induced in rats using the modified Tang's method [18, 19]. Closed head mild traumatic brain injury was produced using a weight drop device. A 175 g steel weight was dropped on the bregma of rats. The drop height was 30 cm and the weight went through a polyvinyl chloride tube (inner diameter 11 mm) to offer regular drop site. Rats were placed on a wooden plate and fixed by Velcro in a prone position. Rats were subjected to three consecutive injuries in a single day.

### 2.3. Anodal tDCS

Anodal tDCS was applied using Phoresor II Auto® (IOMED, Salt Lake City, UT, USA) at an intensity of 0.2 mA and a density of 0.255 mA/cm^2^ for 30 min. Anodal tDCS was applied in a single session. A cup-shaped active electrode (1.0 cm diameter) was positioned on the scalp (0.785 cm^2^ contact area) around the left motor cortex using a high-conductivity fixation cream. A counter electrode (3 × 3 cm^2^-sized rubber pad) was positioned on the ventral thorax and wrapped with a tape [20].

### 2.4. Motor-Evoked Potential Test

MEP was recorded from the tibialis anterior muscle bilaterally. A monopolar uninsulated stainless steel needle electrode was inserted into the belly of the tibialis anterior muscle as an active electrode and into the tendon of the tibialis anterior muscle as a reference electrode. The ground was positioned at the site of the tail origin. A figure-eight-shaped transcranial magnetic stimulation (TMS) coil, Magstim magnetic stimulator® (Magstim Company Ltd., Whitland, Wales, UK), was positioned within the contralateral motor cortex whose center was anterior and lateral to the bregma. TMS intensity was recorded as percent machine output (MO), with 100% corresponding to the maximal amplitude electrical current conducted through the magnetic coil. We set the stimulation intensity to 100% MO and stimulation at a 7 sec interpulse interval. The most large peak-to-peak amplitude and the earliest latency of MEP among the results of at least 10 trials were assessed [21].

### 2.5. Somatosensory-Evoked Potential Test

SEP was recorded from the cortex during tail stimulation. The active electrode inserted 2.5 mm posterior to the bregma and the reference electrode inserted in the mid frontal bone. The ground was placed on the sole of the left hind limb. Electrical stimulation was performed via surface electrodes which were positioned at the site of the tail origin and 4 cm distal area. Peak-to-peak amplitude and P1 latency were averaged over 200 stimulations at a 2.0 mV stimulation intensity [22].

### 2.6. Brain MRI

Rats were anesthetized with an intramuscular injection of Zoletil (tiletamine/zolazepam, 15 mg/kg) and brain MRI was performed at 12 days after rmTBI. MRI scans were performed with a four-element-phased array animal dedicated with a 5 cm inner diameter surface coil (Chenguang Medical Technology Co., Ltd., Shanghai, China). A standard spin echo sequence (TE 22 ms; TR 650 ms; slice thickness 3.00 mm; matrix scan 512; FOV 100.00 mm) was used to acquire the T2-weighted images [19].

### 2.7. Immunohistochemistry

At 12 days after injury, the animals were deeply anesthetized with Zoletil and euthanized. The brains were extracted and fixed by immersion in 10% buffered formalin solution. Serial coronal sections of the brain were obtained, and 5 *μ*m-thick sections including the motor cortex (primary and secondary motor cortex) and the external capsule were prepared for H-E stain and immunohistochemical study with anti-glial fibrillary acidic protein (GFAP) antibody (ab4674, Abcam, Cambridge, MA, 1 : 500 dilution, 30 min) and secondary antibody (ab6877, Abcam, 1 : 200 dilution, 20 min). Immunohistochemical study was performed with Bond Max (Leica Biosystems, Newcastle, UK). Integral intensity of astroglial immunoreactivity in the GFAP staining was measured by computer-assisted image analysis program (AnalySIS, Soft Imaging System, GmbH, Müster, Germany). Images were captured from motor cortex and external capsule. The software automatically changed the color of all immunolabeled elements beyond the threshold range into red pixels and changed the color of the rest of the image into gray pixels. The software then estimated the intensity of pure red pixels [23].

### 2.8. Statistical Analysis

Wilcoxon signed-rank test was performed to confirm the comparisons of measurements between before and after injuries or anodal tDCS. To analyze the differences among the three groups, Kruskal-Wallis test was used. Statistical analysis was performed using SPSS ver. 20.0 (IBM SPSS, Armonk, NY, USA) and *p* values under 0.05 were considered statistically significant.

## 3. Results

There was no serious adverse event after injury and anodal tDCS treatment, and all rats survived the first study day. But, three rats died during anesthesia, which was applied after twelve days for MRI.

### 3.1. Recovery of the Righting Reflex

**Figure 1:**
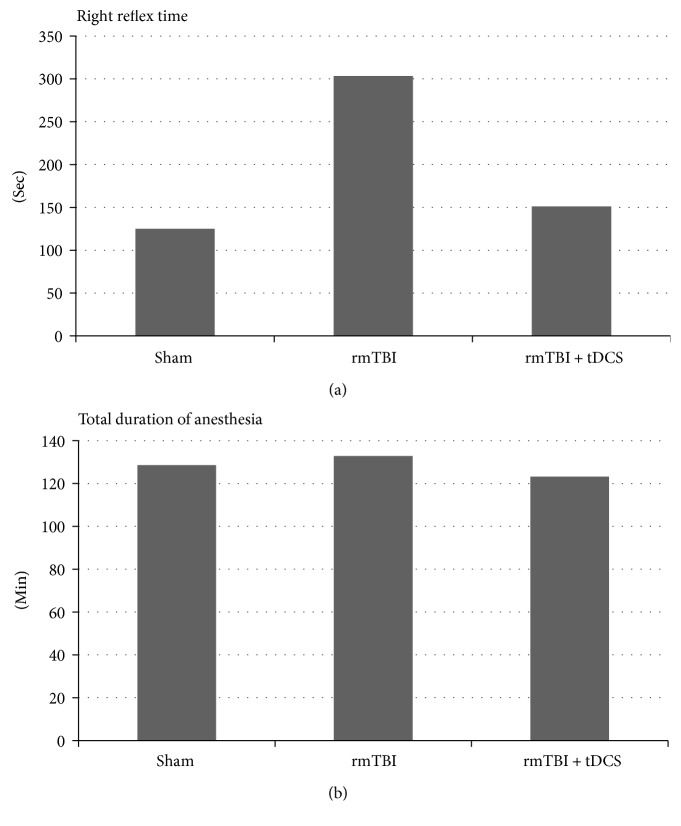
Righting reflex time was increased after repetitive mild traumatic brain injury (rmTBI) compared to that of the sham group, and it was decreased after anodal tDCS therapy (a). Total duration of anesthesia did not change according to brain injury and anodal tDCS therapy (b).

Repetitive mild traumatic brain-injured rats had prolonged recovery time of the righting reflex compared to anodal tDCS-treated group (303.46 ± 181.56 sec versus 151.20 ± 131.46 sec, *p* = 0.049) and sham group (125.00 ± 98.93 sec, *p* = 0.024). There was no significant difference in the righting reflex time between the sham and anodal tDCS groups. Total duration of anesthesia was not different among the three groups (Figure 1).

### 3.2. MEP Findings

Repetitive mild traumatic brain-injured rats had significantly prolonged latency of MEP (6.227 ± 0.233 msec at baseline versus 6.891 ± 0.517 msec after rmTBI, *p* = 0.010) and decreased amplitude of MEP (0.169 ± 0.116 mV at baseline versus 0.076 ± 0.036 mV after rmTBI, *p* = 0.016), which resulted from left motor cortex stimulation. Also, injured rats had significantly prolonged latency of MEP, which resulted from right motor cortex stimulation (6.300 ± 0.232 msec at baseline versus 7.027 ± 0.648 msec after rmTBI, *p* = 0.008). In the anodal tDCS-treated group, MEP amplitude increased from 0.124 ± 0.066 mV to 0.460 ± 0.253 mV (*p* = 0.009) on the left motor cortex stimulation and from 0.151 ± 0.075 mV to 0.406 ± 0.259 mV (*p* = 0.005) on the right motor cortex stimulation. But, the latency after anodal tDCS treatment did not change significantly. The latency and amplitude of MEP in the sham group were not significantly changed on the bilateral motor cortex stimulation. The baseline measurements of MEP were not significantly different among the three groups (Table 1).

### 3.3. SEP Findings

Repetitive mild traumatic brain-injured rats had significantly prolonged P1 latency of SEP (13.85 ± 1.22 msec at baseline versus 14.57 ± 1.11 msec after rmTBI, *p* = 0.022) and decreased amplitude of SEP (1.21 ± 0.34 mV at baseline versus 0.78 ± 0.37 mV after rmTBI, *p* = 0.026), which resulted from tail stimulation. In the anodal tDCS-treated group, the P1 latency and amplitude of SEP did not change significantly (latency 14.50 ± 0.62 msec at baseline versus 14.18 ± 0.85 msec after anodal tDCS treatment, *p* = 0.341; amplitude 1.26 ± 0.47 mV at baseline versus 1.59 ± 1.23 mV after anodal tDCS treatment, *p* = 0.386). The P1 latency and amplitude of SEP in the sham group were not significantly changed. The baseline measurements of SEP were not significantly different among the three groups (Table 2).

### 3.4. MRI Findings

**Figure 2:**
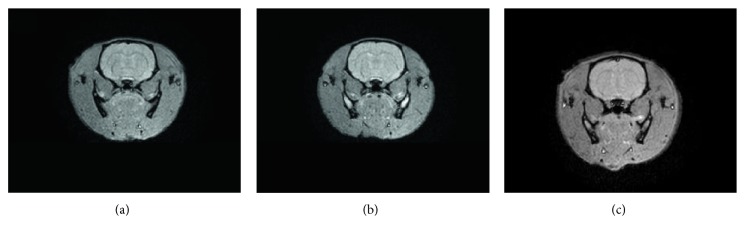
Magnetic resonance imaging findings among rats with sham (a), repetitive mild traumatic brain injury (b), and anodal tDCS (c) were not significantly different.

To assess any overt structural brain damage, we conducted T2-weighted brain MRI for 30 slices from the frontal tip to the brain stem. Repetitive mild traumatic brain injury did not result in significant volumetric changes such as hydrocephalus and cortical thinning. A similar result was observed in the anodal tDCS group and the sham group (Figure 2). There was no fatal injury finding like hemorrhage, diffuse axonal injury, or skull fracture in all animals.

### 3.5. Immunohistochemical Findings

Gross examination of 5 brains in each 3 groups, a total of 15 brains, showed no grossly identified abnormal findings. Lateral ventricles were not grossly enlarged. There was no evidence of neuronal degeneration in all 15 cases on H-E stain.

**Figure 3:**
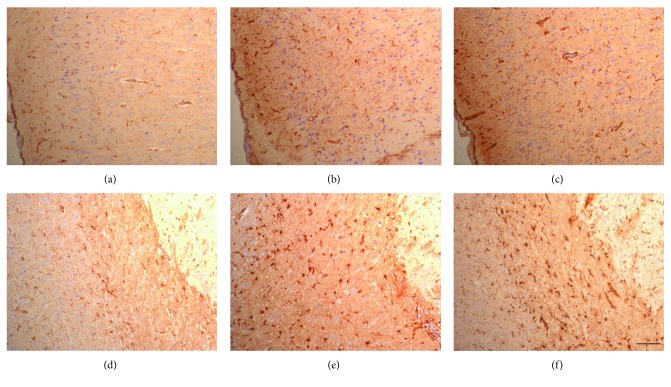
Repetitive mild traumatic brain injury led to a slightly increased GFAP expression in the cortex (b) and external capsule (e) compared with that of the sham group (a and d). The result was not significantly different after anodal tDCS (c and f). The calibration bar represents 200 *μ*m scale.

**Figure 4:**
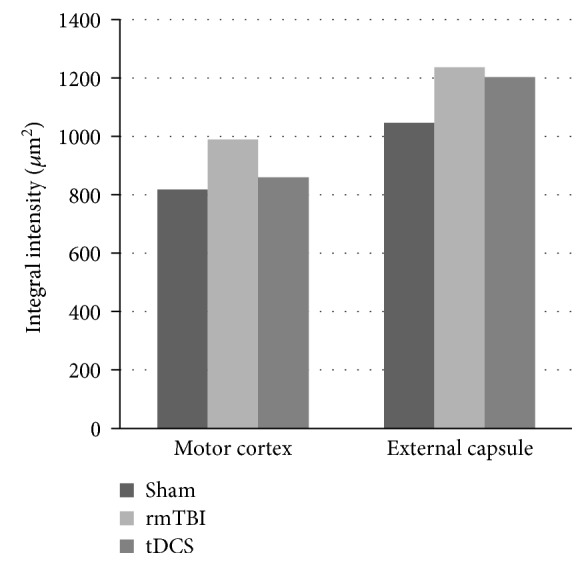
The labeling of GFAP in the motor sensory cortex and external capsule was maximally increased in the repetitive mid traumatic brain injury (rmTBI) group compared to that in the sham and anodal tDCS-treated groups.

According to immunohistochemical study with GFAP stain, hypertrophy of cell body and minimal extension of cell processes were observed in the rmTBI and anodal tDCS groups compared to those in the sham injury group (Figure 3). The integrated intensity of GFAP was measured and calculated in terms of mean values and standard deviations. The integrated intensities of GFAP in the rmTBI and anodal tDCS groups were increased in comparison with that in the sham group. But, the difference was not statistically significant. The integrated intensity of GFAP in the sham group was 818.50 ± 78.49 *μ*m^2^ at the cortex and 1046.94 ± 278.57 *μ*m^2^ in the external capsule. In the rmTBI group, the integrated intensity was 989.36 ± 151.48 *μ*m^2^ at the cortex and 1236.70 ± 95.93 *μ*m^2^ from the external capsule to the caudate putamen. In the anodal tDCS group, the integrated intensity was 859.73 ± 90.94 *μ*m^2^ at the cortex and 1203.45 ± 66.04 *μ*m^2^ from the external capsule to the caudate putamen (Figure 4).

## 4. Discussion

This study demonstrated that rmTBI caused loss of consciousness and affected the electrophysiological results. It also proved that anodal tDCS, administered immediately after brain injury, yielded therapeutic benefits for loss of consciousness and electrophysiological change. The decreased motor and sensory cortical excitability, which was resulted by repetitive mild traumatic brain injury, was restored by tDCS treatment. It could suppose that neural plasticity was induced by tDCS treatment after repetitive mild traumatic brain injury. And it led to early recovery of loss of consciousness.

In animals, the duration of righting reflex could imply the alertness. In this study, rats had prolonged recovery time of the righting reflex after rmTBI, but application of anodal tDCS to rats after rmTBI resulted in earlier recovery of the righting reflex compared to that of repetitive mild traumatic brain-injured rats. Also, the righting reflex time in the anodal tDCS-treated group was similar to that in the sham group. In other words, anodal tDCS has a positive effect on the recovery from loss of consciousness.

The MEP results showed prolonged onset latency and decreased peak-to-peak amplitude after repetitive mild traumatic brain injury compared to the MEP results before injury. After anodal tDCS treatment, the peak-to-peak amplitude of MEP was increased compared to that before injury. Our findings indicate that rmTBI induces a decrease in cortical excitability of the motor cortex and the decreased cortical excitability recovered after anodal tDCS management. A previous study revealed that rmTBI caused behavioral impairment [3–5]. The accumulation and repetition of decreased cortical excitability would extend the behavioral change, and then, anodal tDCS could be a novel management option for behavioral impairment resulting from rmTBI if further long-term study supports our results. A recent study proved that anodal tDCS applied during the early stage of traumatic brain injury had a beneficial effect on behavioral and spatial memory [16]. Traumatic brain injury is not a common disease that is treated by tDCS because of seizure tendency. However, Yoon et al. applied anodal tDCS in traumatic brain injury and showed a therapeutic effect [16]. Similar to Yoon's study, our study showed the potential of anodal tDCS treatment on rmTBI. Furthermore, our low-intensity stimulation method was safe because there was no adverse event such as seizure during anodal tDCS treatment.

The SEP results showed prolonged P1 latency and peak-to-peak amplitude after rmTBI compared to the findings before injury. This finding indicates that rmTBI also induces a decrease in cortical excitability of the sensory cortex. After anodal tDCS treatment, the latency and amplitude of SEP improved as the values of baseline. It indicates that rmTBI induces a decrease in cortical excitability of the sensory cortex, but the decreased cortical excitability could not recover after anodal tDCS treatment. Monai et al. proposed that tDCS changes the metaplasticity of the cortex through increased astrocytic signaling [24]. In other words, tDCS could induce increased neural plasticity of the nondirectly stimulated site. Therefore, the neural plasticity of the sensory cortex could occur even though anodal tDCS stimulated the motor cortex.

MRI findings showed no significant macroscopic brain change in all animals. Wright et al. reported results similar to those of our study. An advanced MRI technique such as tractography detected abnormalities in mild traumatic brain-injured rats compared to sham-injured rats. However, mild traumatic brain injury did not result in significant volumetric changes in any of the brain lesions, including those in the ipsilateral and contralateral cortex, corpus callosum, hippocampus, and lateral ventricle [25]. On the contrary, Goddeyne et al. reported that mild traumatic brain injury resulted in severe ventriculomegaly and cortical thinning [10]. Judging from the previous studies and our study, if the intensity of the impact caused by rmTBI is strong, it can cause ventriculomegaly and cortical thinning. However, when the intensity is weak, it is difficult to observe the change in gross anatomy. We also think that as the number and intensity of rmTBI increase, it will be easier to observe macroscopic brain damage. Therefore, to diagnose rmTBI, an electrophysiological study could be a diagnostic tool that has higher sensitivity than a radiological study such as MRI.

Reactive astrocytosis is the pathological hallmark of central nervous system lesions and gradated continuum of progressive changes [26]. Although rmTBI did not induce a significant increase in astrocytosis compared to that of the sham group, the mean value of GFAP labeling was increased in the cortex and white matter after brain injury and a minimal decrease was observed after tDCS in this study. Throughout the gray matter and white matter, upregulation of expression of GFAP and hypertrophy of cell body were observed after rmTBI and anodal tDCS. It was mild to moderate reactive astrocytosis. But, the expression of GFAP in the injured brain was more excessive compared to that in the brain treated by anodal tDCS. It means that anodal tDCS has a possibility to reduce the severity of brain damage after rmTBI even though significant changes were not defined in this study. The insignificant change in immunohistochemical study was supposed to be caused by the number of anodal tDCS treatments being limited to only once. The effect of multiple anodal tDCS treatments should be revealed in further studies.

The limitation of this study was that tDCS treatment was just applied in a single session. The multiple times of tDCS attempt could induce critical changes in electrophysiological findings and histologic findings. And a small sample size was the limitation to reveal the pathologic change from anodal tDCS. However, this single tDCS treatment for repetitive mild traumatic brain injury and small sample-sized study can provide preliminary data for further studies.

In conclusion, a single anodal tDCS can have a positive effect on repetitive concussion in terms of loss of consciousness and modulation of cortical excitability. Anodal tDCS after rmTBI seems to be a useful tool for promoting transient motor recovery through increasing the synchronicity of cortical firing, and it induces early recovery of consciousness. In the future, the effect of numerous sessions of anodal tDCS therapy on repetitive mild traumatic brain injury could reveal whether it can protect the brain against a delayed unpleasant degenerative change in the brain and functional impairment.

## Figures and Tables

**Table 1:** The result of motor-evoked potential (MEP) in three groups.

	Rt. MEP latency (msec)	Rt. MEP amplitude (mV)	Lt. MEP latency (msec)	Lt. MEP amplitude (mV)
Sham				
Baseline	6.309 ± 0.358	0.131 ± 0.059	6.472 ± 0.371	0.119 ± 0.075
Postinjury	6.364 ± 0.376	0.122 ± 0.071	6.578 ± 0.292	0.092 ± 0.066
rmTBI				
Baseline	6.227 ± 0.233	0.169 ± 0.116	6.300 ± 0.232	0.132 ± 0.068
Post-rmTBI	6.891 ± 0.517^∗^	0.076 ± 0.036^∗^	7.027 ± 0.648^∗^	0.150 ± 0.229
rmTBI + tDCS				
Baseline	6.550 ± 0.272	0.124 ± 0.066	6.650 ± 0.337	0.151 ± 0.075
Post-tDCS	6.409 ± 0.626	0.460 ± 0.253^∗^	6.584 ± 0.737	0.406 ± 0.259^∗^

Values are expressed as mean ± standard deviation. rmTBI: repetitive mild traumatic brain injury; tDCS: anodal transcranial direct current stimulation. ^∗^*p* < 0.05: compared to the result at baseline.

**Table 2:** The result of sensory-evoked potential (SEP) in three groups.

	SEP latency (msec)	SEP amplitude (mV)
Sham		
Baseline	13.75 ± 1.13	0.84 ± 0.38
Post-injury	13.91 ± 1.30	0.98 ± 0.46
rmTBI		
Baseline	13.85 ± 1.22	1.21 ± 0.34
Post-rmTBI	14.57 ± 1.11^∗^	0.78 ± 0.37^∗^
rmTBI + tDCS		
Baseline	14.50 ± 0.62	1.26 ± 0.47
Post-tDCS	14.18 ± 0.85	1.59 ± 1.23

Values are expressed as mean ± standard deviation. rmTBI: repetitive mild traumatic brain injury; tDCS: anodal transcranial direct current stimulation. ^∗^*p* < 0.05: compared to the result at baseline.
